# Laparoscopic resection of cecal duplication cyst in a 28 years old male: a rare case report

**DOI:** 10.1186/s12893-025-03291-z

**Published:** 2025-11-11

**Authors:** Jaafar AL Shami, Georges Gandour, Hussien El Moussawi, Zahraa Adel, Ali Al Harake

**Affiliations:** 1 Department of General Surgery, Lebanese University, Beirut, Lebanon; 2 Department of General Surgery, University of Balamand, Beirut, Lebanon; 3 Department of General Surgery, AL Karameh Teaching Hospital, Baghdad, Iraq

**Keywords:** Duplication cyst, Cecal duplication cyst, Laparoscopic resection, Case report

## Abstract

**Background:**

Cecal duplication cysts are a rare congenital anomaly, particularly uncommon in adults. With no clear etiology behind it, duplication cysts typically appear before 2 years of age. Their presentation is diverse, often mimicking other right lower quadrant pathologies. We report a case of an adult cecal duplication cyst treated successfully with laparoscopic resection, highlighting diagnostic challenges and management considerations.

**Case Presentation:**

A 28-year-old man presented to our care with a one-week history of right lower quadrant pain. Initial ultrasound and CT scan suggested a paracecal cystic lesion, with differential diagnoses including cecal diverticulitis or epiploic appendagitis. Partially responsive to medical therapy, a repeat CT after 10 days was performed and showed an interval enlargement of the lesion with persistent fat stranding. Diagnostic laparoscopy revealed a cystic mass densely adherent to the cecal wall, sharing a common blood supply. Ileocecectomy with side-to-side ileocolic anastomosis was performed. Histopathology confirmed a cecal duplication cyst lined by colonic mucosa with a smooth muscle layer and no evidence of dysplasia or malignancy. The patient recovered uneventfully and remained asymptomatic at 6-month follow-up.

**Conclusion:**

Cecal duplication cysts in adults are exceedingly rare, representing less than 0.5% of gastrointestinal duplications. Diagnosis is challenging due to overlapping clinical and radiologic features with more common conditions. Histopathology remains the gold standard for confirmation. Complete surgical resection is the treatment of choice, both to relieve symptoms and to mitigate risks of complications, including malignant transformation.

## Background

Duplication cyst is a rare congenital abnormality of the gastrointestinal tract that manifests anywhere in the alimentary tract, mainly in the ileum and the jejunum [1]. The majority (67–80%) present before two years of age. They can be divided into two types, either cystic (80%) or tubular (20%), and appear adjacent to or within the alimentary tract [2]. Many hypotheses were postulated regarding the etiology of gastrointestinal duplication cysts, such as recanalization, split notochord, environmental factors, or the presence of embryologic diverticula. However, no theory could reach a definitive conclusion concerning their origin [3]. Colonic duplication cysts are mainly asymptomatic. However, they may lead to possible complications like perforation, obstruction, or bleeding [4]. When it comes to investigations, multiple methods can be opted for to identify colonic duplication cysts, from endoscopic ultrasound to CT scan, or contrast enema. In addition, those communicating with the colonic wall may possibly be identified through colonoscopy without a total guarantee of success [5].

In fact, duplication cysts are most commonly diagnosed intraoperatively, although there is difficulty in differentiating them from other mesenteric masses. One group of surgeons may prefer to surgically excise only the symptomatic cases, whereas the second would stand with resection of the cyst whenever diagnosed to avoid potential complications or risk of neoplastic transformation [6]. In our study, a duplication cyst was considered in the differential diagnosis plan based on the radiological findings, but was not confirmed until the pathology report came post-operation. Our case represents one of the rare locations of a duplication cyst, the cecum, with an atypical presentation, and treated by laparoscopic resection.

## Case presentation

A 28-year-old Caucasian male patient, previously healthy, presented with a one-week history of right lower quadrant (RLQ) pain. Pain is qualified as stabbing in nature, increases with motion, intermittent, with no radiation to other areas. The patient did not report any other symptoms, and a history of previous trauma or urinary problems was negative.

Vital signs on presentation were as follows: blood pressure 110/70 mmHg, heart rate 74 beats per minute, respiratory rate 13 breaths per minute, temperature 36.6 Celsius (°C), and an oxygen saturation of 98%. Physical examination revealed a soft abdomen with no visible or palpable mass, associated with mild tenderness upon deep compression on the RLQ. Respiratory, cardiovascular, and neurological exams were unremarkable. Routine laboratory tests on admission were normal regarding complete blood count, C-reactive protein, and urine analysis. Abdominopelvic ultrasound was ordered and revealed a small paracecal cystic collection of 2 × 2 cm without signs of appendicitis or free fluid. Thus, a CT scan of the abdomen and pelvis with IV and PO contrast was requested, demonstrating a 2.3 × 1.8 cm cystic collection with internal septation at the level of the cecum, 4 cm above the normal appendix, with mild fat stranding. The two major differential diagnoses at this level, based on the clinical presentation and the radiological findings, were cecal diverticulitis or complicated appendagitis. After consultation with the gastroenterology team, a 10-day course of Ibuprofen 400 mg orally twice daily, Ciprofloxacin 500 mg orally twice daily, and metronidazole 500 mg orally three times daily was started.

**Fig. 1:**
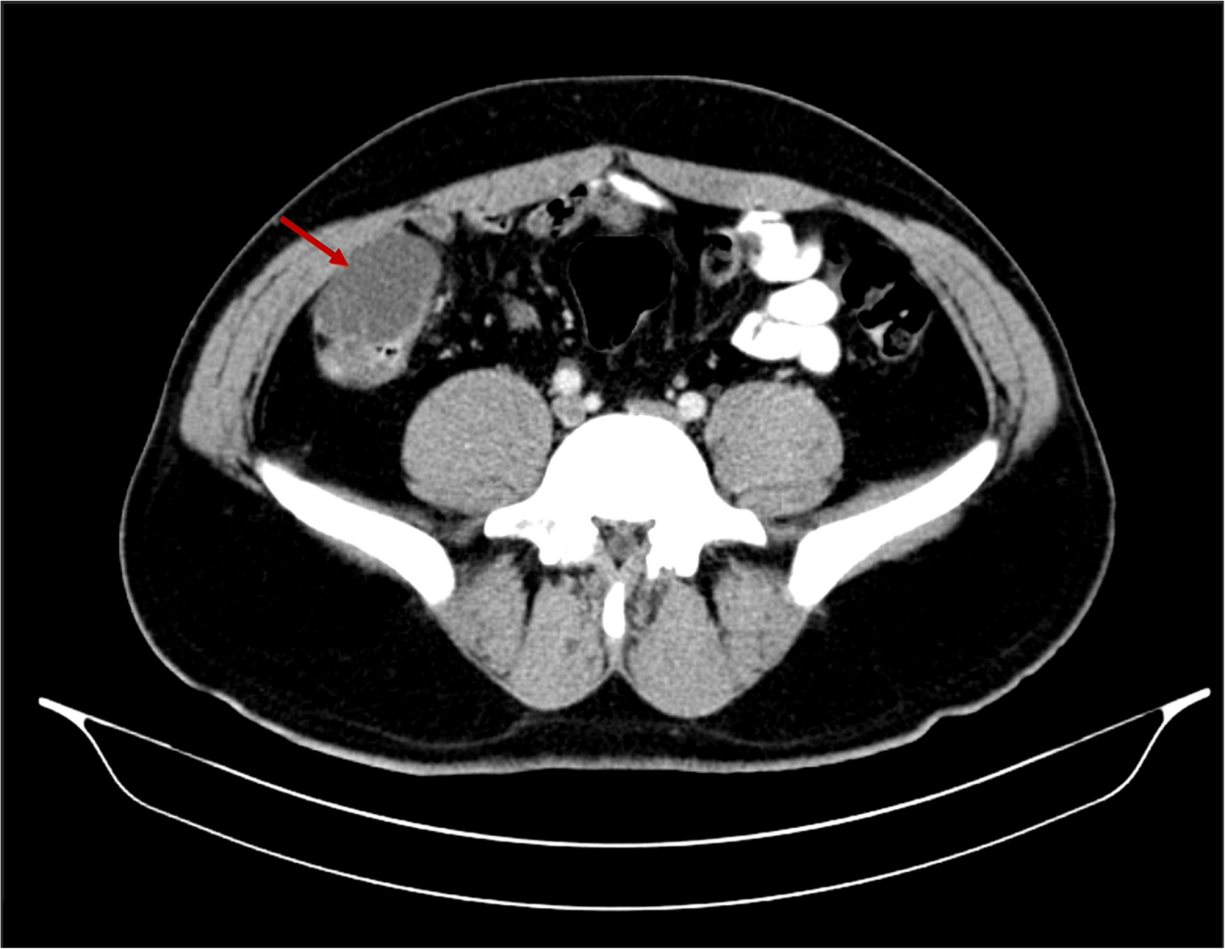
Cystic collection to 4.3 × 5.5 cm, with fat stranding and adherence to the cecal wall medially and the abdominal wall laterally

**Fig. 2:**
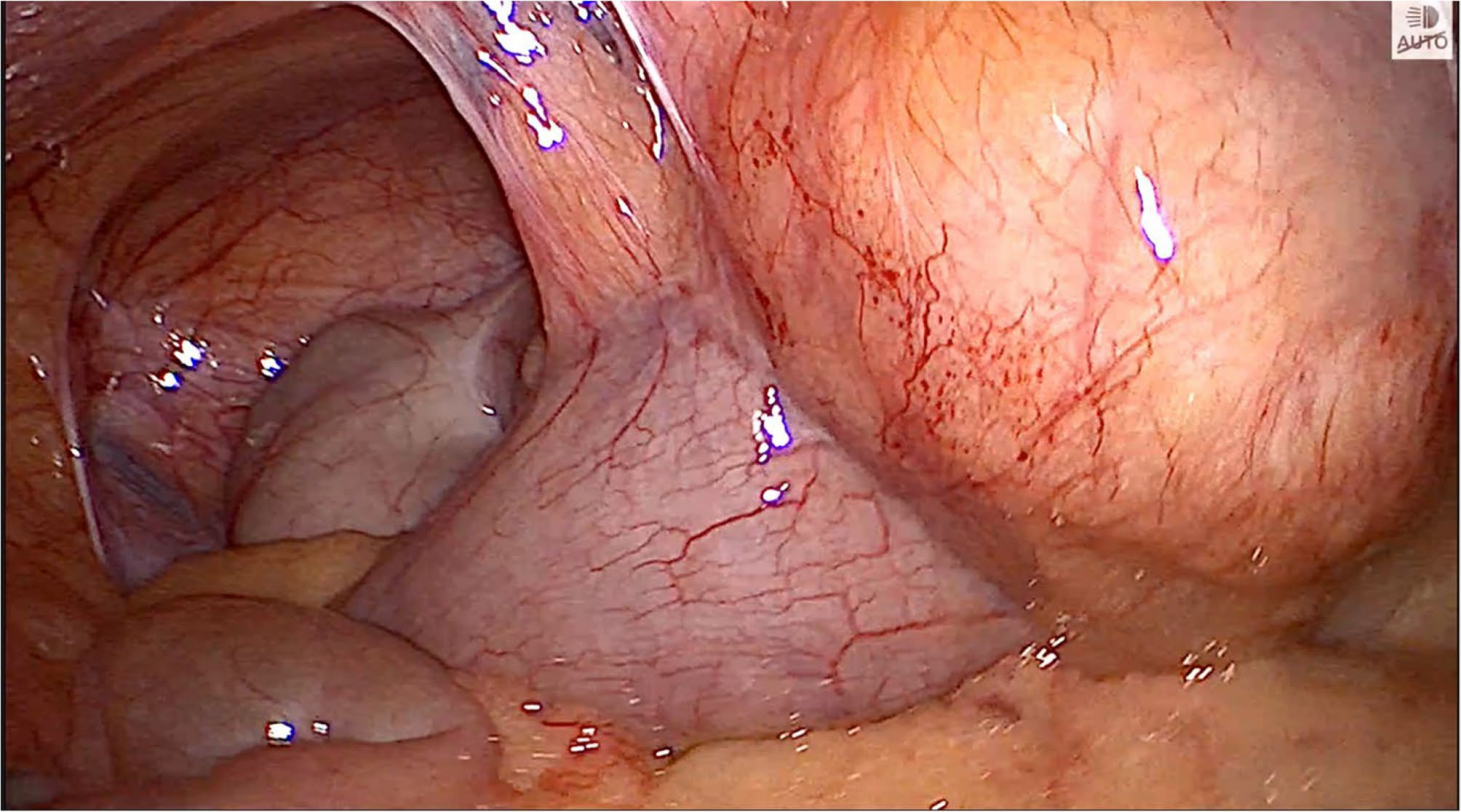
Cecal mass severely adherent to the cecal wall and to the lateral peritoneal reflection

**Fig. 3:**
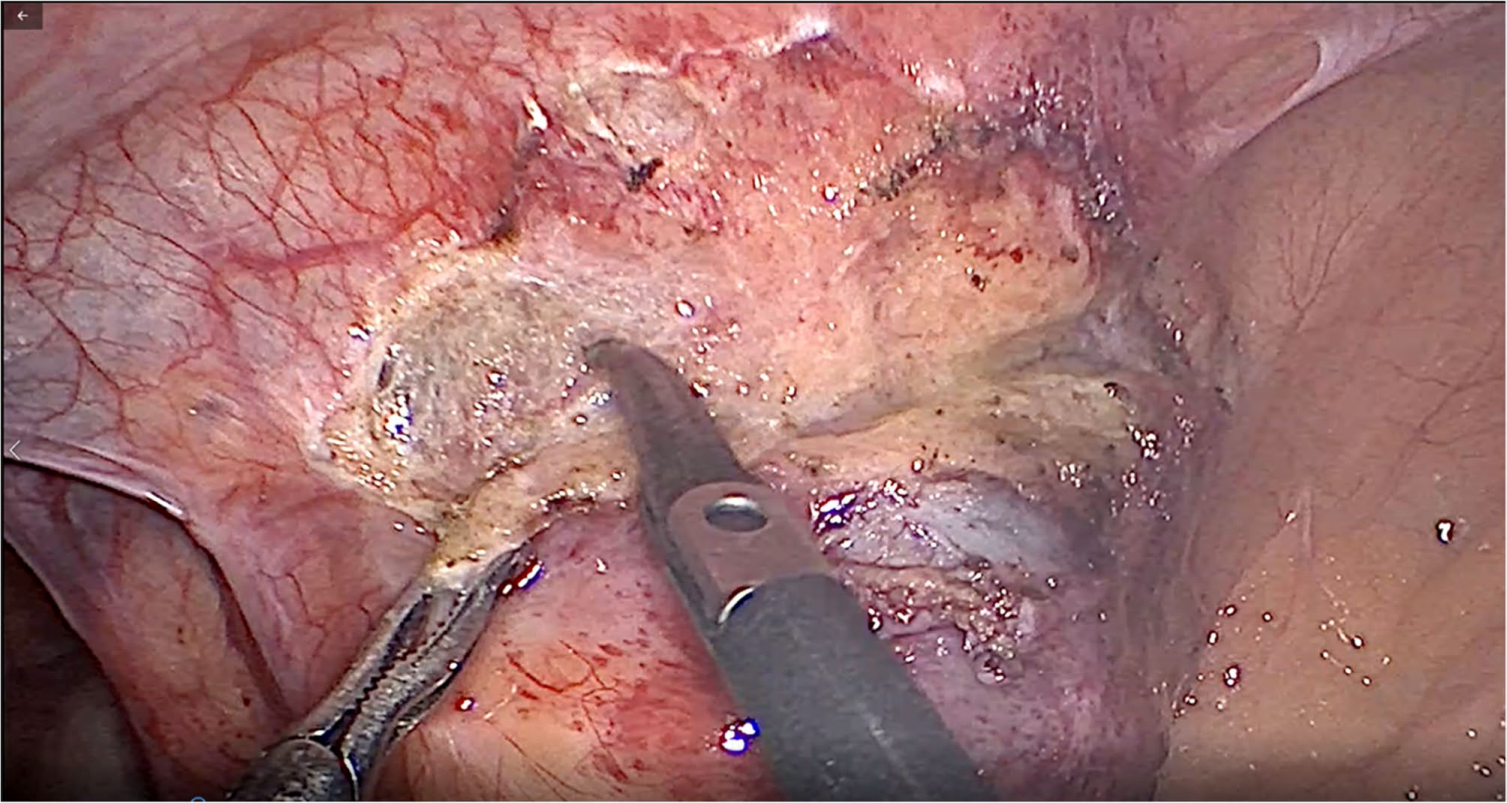
Dissection of the mass from the lateral wall using scissors and cautery

**Fig. 4:**
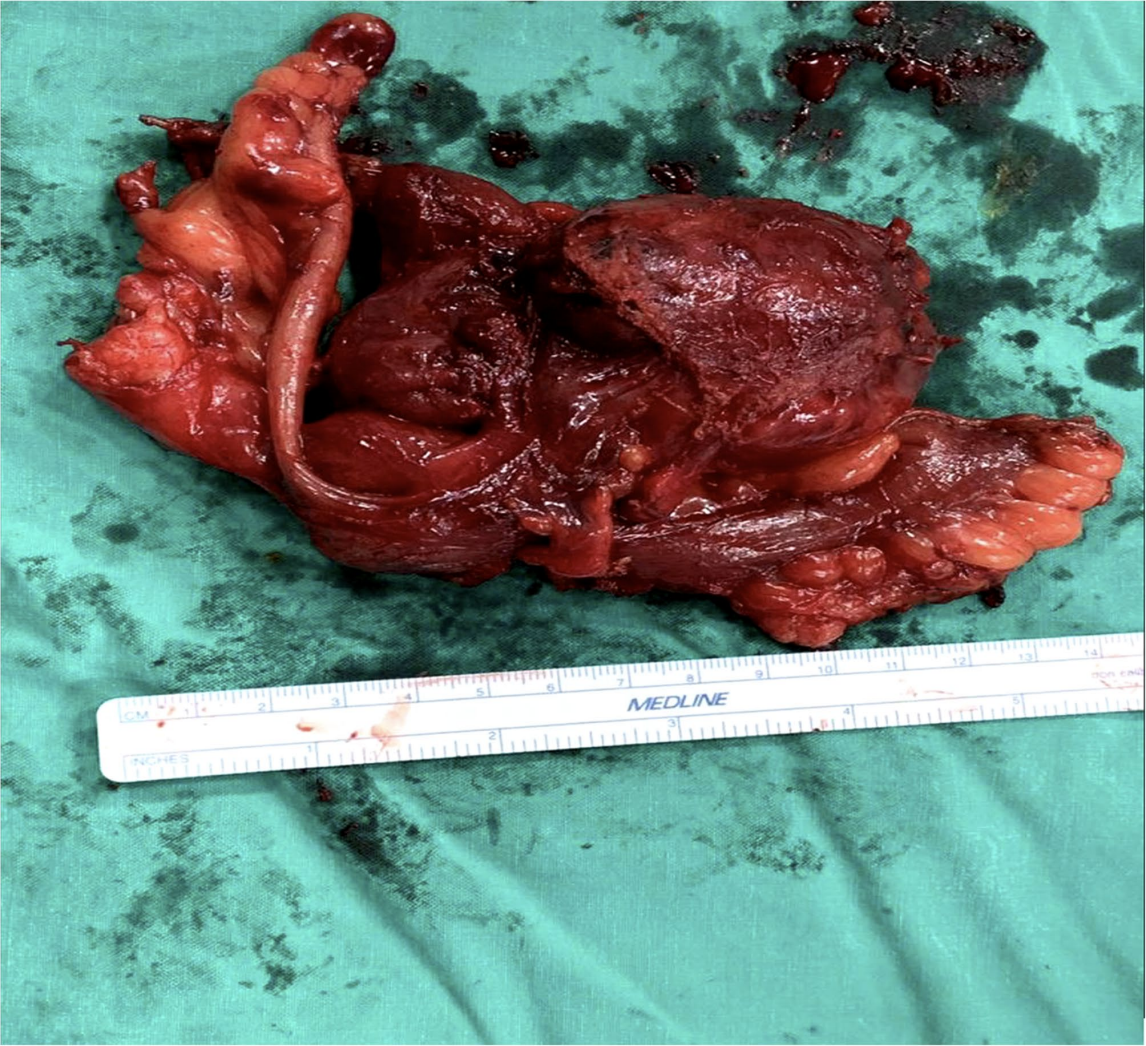
Specimen of the ileum, cecum, and cyst

Upon reassessment post 10-day course, the patient stated that the pain decreased in severity but did not go away and was still causing discomfort. A follow-up abdomino-pelvic CT with IV and PO contrast was performed and revealed an increase in the size of the cystic collection to 4.3 × 5.5 cm, with enhancement of the previous fat stranding, adherence to the cecal wall medially, and to the abdominal wall laterally, with the suggestion of a possible complicated appendagitis or duplicated cyst as the main cause (Fig. 1). No mass lesion, adenopathy, or ascites was noted. The decision was taken to go for diagnostic laparoscopy. After insufflation and pneumoperitoneum creation, a fixed mass was perceived on the cecum, severely adherent to the cecal wall, and to the lateral peritoneal reflection, which was inconsistent with inflammatory lesions such as diverticulitis or epiploic appendagitis (Fig. 2). Successful dissection of the mass from the lateral wall was accomplished using scissors and cautery (Fig. 3). However, upon dissecting the mass from its connection to the cecal wall, great difficulty was experienced, with failure of the dissection, especially after noticing the presence of a common vessel supplying the cecum and the cyst at the same time. As a result, an ileocecectomy was decided by exteriorizing the ileum, cecum, and the cyst through a 6 cm RLQ incision and applying a mechanical side-to-side ileocolic anastomosis, then returning the bowel to the peritoneal cavity, followed by deflation and closure (Fig. 4). Postoperatively, the patient had mild pain with no major complaints, and was discharged home four days later.

The pathology report came back as a cystic lesion measuring 5 × 4 × 2.8 cm, adherent to the cecal serosal tissue with no communication to the cecal lumen, alongside the presence of duplication of the colonic wall at the cecal level, spherical in type, thus confirming the diagnosis of a cecal duplication cyst as the root etiology of our patient’s condition. No granuloma or malignancy was observed. The patient remains asymptomatic at the 6-month outpatient follow-up appointment.

## Discussion

Enteric duplication cysts are a rare congenital anomalies. They can originate anywhere in the gastrointestinal tract, with the small intestine being most commonly involved, mainly in its distal part [7]. Out of the duplication cysts, only 13% are present in the colon [8]. In fact, cecal duplication cysts are rare and comprise only 0.4% of all gastrointestinal duplications [9]. In a review made by Oudshoorn regarding 362 cases of duplication cysts, only 16 patients had Cecal duplication cysts (CDC) [10]. Thus, we can highlight the importance of our case, reporting a duplication cyst in the cecum. In terms of age, CDC may arise at any age, with the majority being discovered within 2 years after birth [11]. They are also more common in females, which goes opposite to our case, as our patient is a 28-year-old male, atypical in both age and gender. [12]

Duplication cysts may be divided into two types: tubular or spherical. The spherical type is the more common presentation and corresponds to our patient’s diagnosis. Presenting features may differ depending on the site, size, and morphology of the CDC. However, the common symptoms and signs are vomiting, palpable abdominal mass, abdominal distension, abdominal pain, blood in stools, and constipation [13]. Our patient only complained of persistent abdominal pain mainly in the right lower quadrant, with no other associated symptoms.

To continue, it is crucial to understand that duplication cysts may have a malignant transformation, and cases of adenocarcinoma from the cyst wall have been reported [14]. When it comes to investigations, ultrasound is the most common modality used for the diagnosis, with the ‘double-wall’ sign being highly suggestive of enteric duplication in terms of both high specificity and positive predictive value. CT and MRI are less commonly used as modalities when a duplication cyst is suspected [12]. In our case, the abdominopelvic CT scan with IV and PO contrast was ordered due to persistent pain over several days, and to assess the visceral organs, mainly the bowels and surroundings, in a more precise manner, especially since the patient was overweight. The scan came back positive for a cystic cecal mass, and due to no improvement despite medical treatment, another follow-up control CT scan was performed to assess the previous findings. At this stage, a great radiological suspicion of a duplication cyst was raised.

Furthermore, duplication cysts share common characteristics with the gastrointestinal tract, such as the blood supply, epithelial lining, and well-developed smooth muscle layer. For small cystic or short tubular duplication, treatment is segmental surgical resection with a primary end-to-end anastomosis. If the lesion cannot be excised to preserve the length of the bowel, as in the case of long tubular cysts, other options can be considered, including marsupialization, enucleation, and mucosal excision [10, 13]. In our case, although the risk of malignancy plays a major role in surgical decision-making, the decision to opt for a segmental resection of the ileocecal segment depended not only on the high suspicion of intraoperative findings of a duplication cyst and its malignant potential , but also due the failure to dissect the cyst from the cecal wall, and after noticing the presence of a common blood supply.

## Conclusion

The CDC may present differently depending on the age of occurrence. One should think of CDC in diagnosing neonates and children presenting with acute or subacute intestinal obstruction. For the imaging modalities used to diagnose duplication cysts, the most common are ultrasonography and abdomino-pelvic CT. However, it is still difficult to locate exactly where the cyst is found. Laparoscopy plays a crucial role in the process of diagnosis and management. The gold standard choice of treatment is complete surgical resection, with the importance of early intervention to prevent extreme morbidities such as sepsis, peritonitis, and perforation. Furthermore, histopathology is the gold standard for a definitive diagnosis of a duplication cyst. This article represents a unique case of a rare anatomical and clinical presentation of a gastrointestinal duplication cyst that was treated by segmental resection with a laparoscopic surgical technique. Future reports and studies with detailed imaging, pathology, and long-term follow-up will be valuable to better characterize the presentation and outcomes of adult duplication cysts.

## Data Availability

All data generated or analysed during this study are included in this published article.

## Abbreviations

CT: Computed Tomography
IV: Intravenous
PO: Per os
RLQ: Right lower quadrant
CDC: Cecal Duplication Cyst
